# *Butyricimonas faecihominis*: an atypically resistant bacterium implicated in abscess formation - a case report

**DOI:** 10.1186/s12879-024-09604-6

**Published:** 2024-07-14

**Authors:** Lukas Wessendorf, Tobias Brezina, Fabian Dusse, Pia Wiegel, Alessa L. Boschert

**Affiliations:** 1 Department of Anesthesiology and Intensive Care Medicine, Faculty of Medicine and University Hospital Cologne, University of Cologne, Cologne, Germany; 2 Institute for Medical Microbiology, Immunology and Hygiene, Faculty of Medicine and University Hospital Cologne, University of Cologne, University Hospital Cologne, Goldenfelsstr. 19 -21, 50935 Cologne, Germany

**Keywords:** Blood stream infection, Abscess, Antimicrobial resistance, Sepsis

## Abstract

**Background:**

This case report presents a unique instance of abscesses with an uncommon pathogen isolated from blood cultures.

**Case presentation:**

We present the case of a perianal abscess in a 50-year-old man with a history of cocaine abuse and bilateral hip replacements. The rapid progression led to septic shock and multi-organ failure, requiring intensive care unit admission, surgery including protective transversostomy. Blood cultures showed growth of *Butyricimonas spp*. with resistance to penicillin and piperacillin-tazobactam. The immediate switch to meropenem led to a significant improvement in the patient’s condition. The patient was discharged after 40 days of hospitalization in good general condition and the reversal of the transversostomy was performed six months later.

**Conclusion:**

The identification of *Butyricimonas faecihominis*, a rarely reported pathogen, emphasizes the challenges of diagnosing and treating unusual infections. This case emphasizes the importance of rapid microbiological diagnosis, interdisciplinary collaboration, and targeted antibiotic therapy in the treatment of abscesses and sepsis.

## Background

Abscesses represent a prevalent clinical presentation stemming from bacterial infections, necessitating prompt surgical intervention and tailored antimicrobial therapy, as untreated abscesses, especially perianal abscesses, can lead to complications such as fistula formation and, rarely, sepsis. However, with further progression, the risk of developing from an encapsulated localized process [1] to non-encapsulated phlegmons increase the likelihood of systemic complications, such as sepsis, and the risk of fatal outcomes [2]. This underscores the importance of expeditiously identifying and characterizing the bacterial pathogen along with its susceptibility profile. However, the specific identification of uncommon or atypical pathogens poses challenges due to the need for specialized laboratory techniques and comprehensive databases for their discernment, compounded by limited clinical familiarity with such pathogens. A considerable proportion of abscesses are attributed to Staphylococcus aureus [3], thus prompting the selection of empirical therapy tailored to this bacterial spectrum. We present a case of abscess formation correlated with the isolation of *Butyricimonas faecihominis* exhibiting uncommon antibiotic resistance in blood cultures. To the best of our knowledge, this is the first described case of *Butyricimonas faecihominis* causing a bloodstream infection in which antibiotic resistance has been detected.

### Case presentation

In January 2024, a 50-year-old man with a history of cocaine abuse and bilateral hip prostheses was admitted to the anaesthetic intensive care unit of the University Hospital Cologne, Cologne, Germany, with a seven-day history of pain in the perianal region progressing into general lower abdominal and testicular pain. Apart from being an active drug user, no other risk factors for abscess development, such as obesity, diabetes mellitus, or trauma to the perianal region, were present. Initial imaging revealed a perianal abscess extending into the ischioanal fossa with scrotal involvement. The patient deteriorated rapidly, developing septic shock with multi-organ failure, necessitating intensive care unit (ICU) admission. Multi-organ failure presented with prerenal kidney failure with severely elevated retention parameters (Creatinine 6.3 mg/dL [reference R: 0.50–1.10 mg/dL], Potassium 6.1mmol/L [R: 3.6–4.8 mmol/L]), markedly elevated inflammatory markers (CRP 526 mg/L [R: < 5.0 mg/L], Procalcitonin 5.37 µg/L [R: < 0.10 µg/L], Leukocytes 44.5 × 10^9^/L [R: 4.40–11.30 × 10^9^/L]), elevated transaminases (ASAT 117 U/L [R: < 50 U/L], ALAT 72 U/L [R: < 50 U/L]) and severe respiratory restrictions with additional oxygen demand.

### Clinical course

After admission to the ICU two sets of peripheral blood cultures were collected. Surgical drainage of the abscess was promptly performed. A protective tranversostoma to safeguard the perianal wound was established. Piperacillin-tazobactam was selected as empirical antibiotic therapy. Retroperitoneal abscesses diagnosed in subsequent CT-Scans followed by interventions and surgery. Vacuum-assisted closure (VAC) therapy was initiated for the management of the wound. Upon admittance, two sets of blood cultures, comprising of one aerobic and anaerobic bottle each, were taken. Within just three days, we were able to detect an unusual growth of *Butyricimonas spp*. that was resistant to penicillin and piperacillin-tazobactam. The microbiologists reacted immediately and informed the attending physicians directly about these significant results and an interdisciplinary assessment of the patient’s clinical situation was carried out. A joint decision was made to switch the anti-infective therapy from piperacillin-tazobactam to meropenem. The patient quickly benefited from the change in antibiotic therapy. Catecholamine support was swiftly tapered and discontinued, alongside a decrease in fluid requirement. Within 24 h of initiating therapy, both inflammatory and renal function parameters exhibited a significant reduction, facilitating the patient’s transfer from the ICU to the general ward. Two further sets of blood cultures taken, remained sterile. Procalcitonin (0.50 µg/L [R: < 0.10 µg/L] after 5 days) and CRP (231.0 mg/L [R: < 5.0 mg/L] after 5 days) decreased significantly. Over the course of hospitalization, eleven surgical procedures and one interventional radiological treatment were conducted. VAC-therapy ceased after 30 days, leading to closure of the scrotal wound. The patient left the hospital 40 days after admission in good general condition. The patient attended the surgical outpatient clinic several times for wound checks and the tranversostoma reversal was performed in July of 2024.

### Microbiological diagnostics

**Fig. 1:**
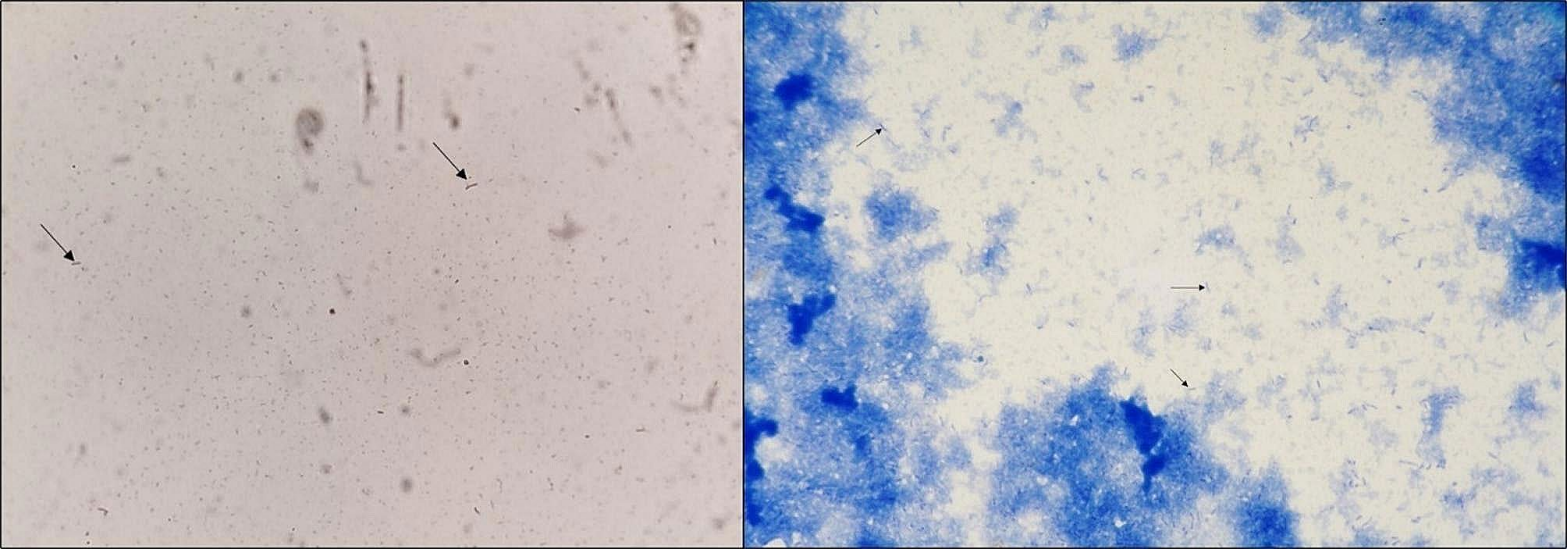
Gram stain (left) and methylene blue stain (right) of the positive blood culture bottle

Blood cultures were automated incubated with the Bactec FX Blood Culture system (Beckton Dickinson, Heidelberg, Germany). After 47 h of incubation one anaerobic bottle became positive, while the other bottles of the initially taken blood cultures – including the second anaerobic bottle - remained sterile. Fine, Gram-negative rods were visible in the subsequently performed Gram stain (Fig. 1). All other blood culture bottles remained negative throughout the incubation period of six days.

**Fig. 2:**
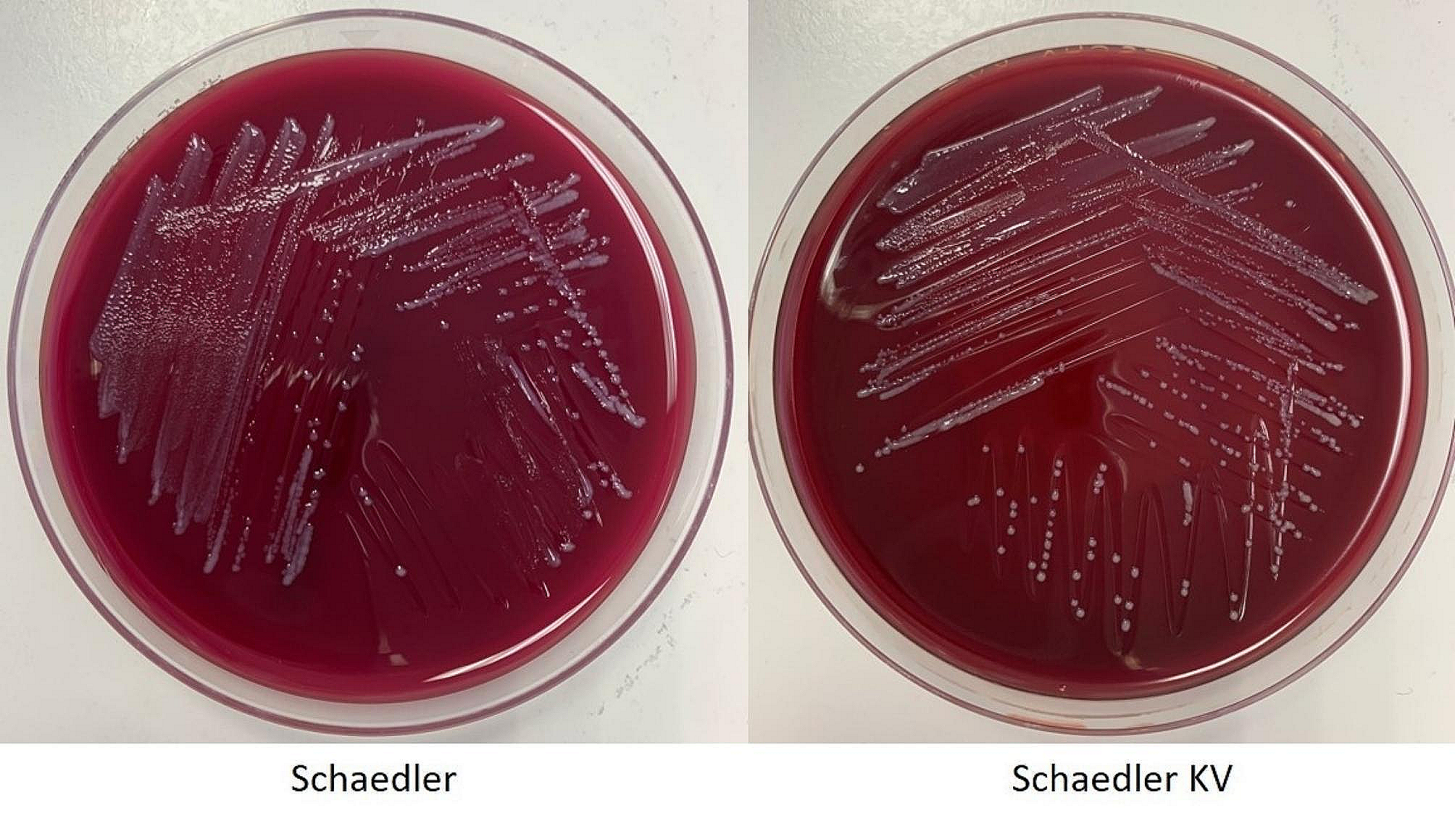
Growth of *Butyricimonas faecihominis* on both anaerobic plates – Schaedler (left) and Schaedler KV (right)

Material from the positive bottle was then streaked on Sheep Blood agar, MacConkey agar, Chocolate agar, Columbia agar, as well as on the two anaerobic agar plates – Schaedler and Schaedler KV agar. While no growth occurred on the aerobic plates, colonies were visible on both anaerobic agar after another incubation of two days (Fig. 2).

The growing organism could be identified as *Butyricimonas virosa* via Matrix-Assisted Laser Desorption/Ionization Time-of-Flight (MALDI-ToF). However, later genome sequencing revealed it to be *Butyricimonas faecihominis*. So far, *Butyricimonas virosa*, albeit rarely, has been reported as the main species of *Butyricimonas spp*. to cause blood stream infections [4]. Yet, our findings suggest that the two species might be prone to be misidentified by mass spectroscopy.

**Fig. 3:**
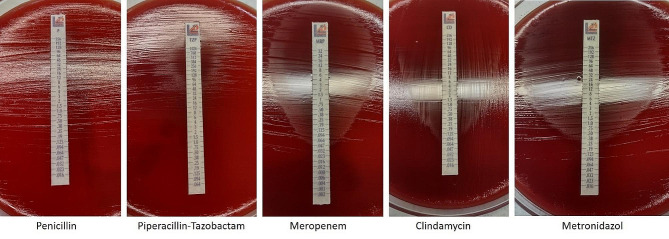
Etest (Epsilometer tests) of penicillin, piperacillin-tazobactam, meropenem, clindamycin, metronidazole (left to right) performed for *Butyricimonas faecihominis*

Antimicrobial susceptibility testing was performed in accordance with the standards provided by the European Committee of Antimicrobial Susceptibility Testing (EUCAST). Unexpectedly, the strain was resistant to both, Penicillin (MIC: > 256 mg/L) and piperacillin-tazobactam (MIC: 128 mg/L) (Fig. 3). Therefore, antimicrobial therapy was adapted from piperacillin-tazobactam to meropenem.

In addition, swabs and biopsies of the abscess were sent for further microbiological investigation. Although anaerobes were cultivated in several samples, *Butyricimonas faecihominis* could not be identified in either of them. This is most likely due to two main reasons. First of all, in several of the samples a high number of anaerobes were found, rendering it impossible to obtain a pure culture of each of them. Secondly, anaerobic bacteria often are highly vulnerable to higher oxygen levels. Hence, not all strains might have remained viable during the transport to the laboratory. Still, the extended perianal abscess has to be considered as the origin of the described bacteremia.

This also emphasizes the importance of rapid transport of critical patient samples to the microbiological laboratory in order to ensure optimum cultural diagnostics.

## Discussion and conclusions

This case underscores the clinical challenges posed by the presence of a rare pathogen, such as *Butyricimonas faecihominis*. This recently discovered species has been isolated from human faeces [5, 6] and has been described as a cause of bloodstream infection [7]. This is also in accordance with our assessment of *Butyricimonas faecihominis* being the potential causative agent for the described case: Pathogens cultivated from abscesses are part of the respective physiological flora [8], hence – in our case - the gastrointestinal microbiota. Furthermore, the patient improved rapidly after switching to a targeted antibiotic therapy.

The significant decrease in Procalcitonin also underlines the therapeutic success: Procalcitonin, as well as the delta neutrophil index (DNI), are potential markers of exudative inflammations. They can also be used to enhance differentiation between bacterial caused inflammation and those of other origin [9, 10]. However, routine infection diagnostics may not include both laboratory parameters. In our case, for instance, DNI is not routinely available.

While other species of *Butyricimonas* have been associated with various infections [11–13], data on antimicrobial susceptibility is limited. Hence, the issues associated with these rare species are even further aggravated, if the pathogens exhibit unexpected antibiotic resistances – as in this case against Penicillin and piperacillin-tazobactam. This stresses not only the pivotal importance of blood culture diagnostics in severely ill patients but also the necessity of tailored antibiotic therapy guided by susceptibility testing. Additionally, our case also underlines the necessity of taking several blood cultures: Only one out of two anaerobic bottles became positive. This might be due to several reasons: Firstly, anaerobic bacteria are fastidious and might hence not survive the taking of the blood cultures and the transport to the laboratory. This is further aggravated by the fact that even in severe bacteremia the bacterial count in the blood is estimated to range between 1 and 10 colony forming units (CFU) per mL [14]. Considering these facts, the detection of potential pathogens is severely hampered by taking only one set of blood cultures.

The swift response to microbiological diagnosis, followed by prompt escalation of antibiotic therapy and subsequent amelioration in the patient’s clinical status, underscores the criticality of close collaboration between microbiologists and attending physicians in expediting time-sensitive treatment decisions and ensure the best possible patient’s outcome.

Additionally, the clinical relevance of a sample might not always be fully apparent from the provided information: In this case, further imaging revealed the full significance of the perianal abscess as a potential source of systemic infection. The latter aspect is also important for clinicians to consider. Providing detailed clinical information about the patient when sending samples for microbiological diagnostics helps to ensure an adequate microbiological diagnostic of the clinical specimen is conducted. The successful treatment of sepsis, which is associated with significant mortality, enabled the patient to be discharged from hospital in good health.

In summary, the rapid identification of an unusual pathogen not typically associated with the usual flora of wound infections and sepsis and the analysis of its antibiotic resistance were crucial. Prompt and effective collaboration between the diagnostic and treatment teams, supported by interdisciplinary assessment and targeted adjustment of antibiotic therapy, contributed significantly to the rapid improvement in the patient’s condition.

## Data Availability

Further information and materials are available from the corresponding author upon reasonable request and in accordance with the principles of medical confidentiality.
